# FilterformerPose: Satellite Pose Estimation Using Filterformer

**DOI:** 10.3390/s23208633

**Published:** 2023-10-22

**Authors:** Ruida Ye, Lifen Wang, Yuan Ren, Yujing Wang, Xiaocen Chen, Yufei Liu

**Affiliations:** 1 Department of Aerospace Engineering and Technology, Space Engineering University, Beijing 101416, China; yerd0103@aliyun.com (R.Y.); wanglifen_2009@139.com (L.W.); faye_liu@aliyun.com (Y.L.); 2 Department of Basic Course, Space Engineering University, Beijing 101416, China; wang-yujing@foxmail.com; 3 Beijing Institute of Special Electromechanical Technology, Beijing 100191, China; xiaocen_88@sina.cn

**Keywords:** satellite pose estimation, pose regression network, filter-based transformer encoder, FilterformerPose

## Abstract

Satellite pose estimation plays a crucial role within the aerospace field, impacting satellite positioning, navigation, control, orbit design, on-orbit maintenance (OOM), and collision avoidance. However, the accuracy of vision-based pose estimation is severely constrained by the complex spatial environment, including variable solar illumination and the diffuse reflection of the Earth’s background. To overcome these problems, we introduce a novel satellite pose estimation network, FilterformerPose, which uses a convolutional neural network (CNN) backbone for feature learning and extracts feature maps at various CNN layers. Subsequently, these maps are fed into distinct translation and orientation regression networks, effectively decoupling object translation and orientation information. Within the pose regression network, we have devised a filter-based transformer encoder model, named filterformer, and constructed a hypernetwork-like design based on the filter self-attention mechanism to effectively remove noise and generate adaptive weight information. The related experiments were conducted using the Unreal Rendered Spacecraft On-Orbit (URSO) dataset, yielding superior results compared to alternative methods. We also achieved better results in the camera pose localization task, indicating that FilterformerPose can be adapted to other computer vision downstream tasks.

## 1. Introduction

Satellite pose estimation is a much needed space detection technology, holding significant application value in spacecraft formation flying (SFF) [1], relative navigation [2], OOM [3], and active debris removal (ADR) [4]. Based on its light mass, small volume, and low power consumption monocular camera [5], space object sensing technology has lower requirements on the payload satellite and is more in line with the special on-orbit space environment. Simultaneously, detection technology based on RGB images offers distinct advantages, including high efficiency and strong robustness, which have garnered considerable attention from research institutions and scholars [6,7,8,9,10]. However, pure vision-based satellite detection encounters many technical issues that require attention, such as the limitations imposed by the lighting environment in space on camera imaging and the relationship between a satellite’s spatial position information and its rotational characteristics. These issues pose many challenges to devising purely vision-based satellite exploration solutions. 

Non-cooperative object pose estimation based on RGB images can be categorized as a six-degrees of freedom (6-DoF) pose estimation problem in computer vision tasks. With the rapid development of deep learning, 6-DoF pose estimation algorithms [11,12,13,14] based on deep learning techniques have gained widespread adoption. Compared with traditional 6-DoF pose estimation approaches, the use of deep learning algorithms can improve model efficiency and robustness. Currently, the 6-DoF pose estimation tasks can be categorized into two types based on their processing flow. In order to accurately estimate the 6-DoF of an object, we typically follow two different methods. In the first method, the key points of the object are first detected, and then, the translation information of the object is determined by perspective-n-points (PnP) and random sample consensus (RANSAC) [12,15]. The second method is based on end-to-end pose estimation [16,17]. This method involves feeding images into the model, which then directly infers pose information. It utilizes only the input image and estimates the object pose with a single forward pass, resulting in significantly lower latency, but at the expense of reduced inference accuracy. In satellite pose estimation missions, end-to-end pose estimation methods should be employed whenever possible to mitigate computational overhead and inference efficiency.

The Spacecraft Pose Network (SPN) [18] is one of the first satellite pose estimation methods that utilized deep learning and RGB images for spacecraft pose estimation. It uses a CNN basic network, constructs three different branches, performs spacecraft 2D bounding box extraction using RCNN and pose classification using a fully connected layer; and then, it obtains N candidate terms through branch 2, using another cross-entropy loss minimization and obtains the relative weight of each candidate; finally, it uses quaternion averaging to obtain the final fine pose. PoseCNN [19] uses a multilayer convolutional network to learn both object translation information and orientation information. This method extracts feature maps at different scales to obtain semantic information from the image, thereby pinpointing the object’s center and predicting translation details. Subsequently, it employs regression to estimate the object’s orientation. Peng S et al. [20] proposed a PVNet algorithm, which identifies key points through pixel-level vector regression. It then uses the RANSAC method to vote for these key locations, effectively addressing occlusion challenges. However, it is worth noting that this algorithm relies on pixel-level features and the RANSAC method, which can result in extended computation times. However, in satellite pose estimation missions, the imperative is to conserve computational resources, shorten inference time, and streamline detection processes as much as possible. 

**Figure 1:**
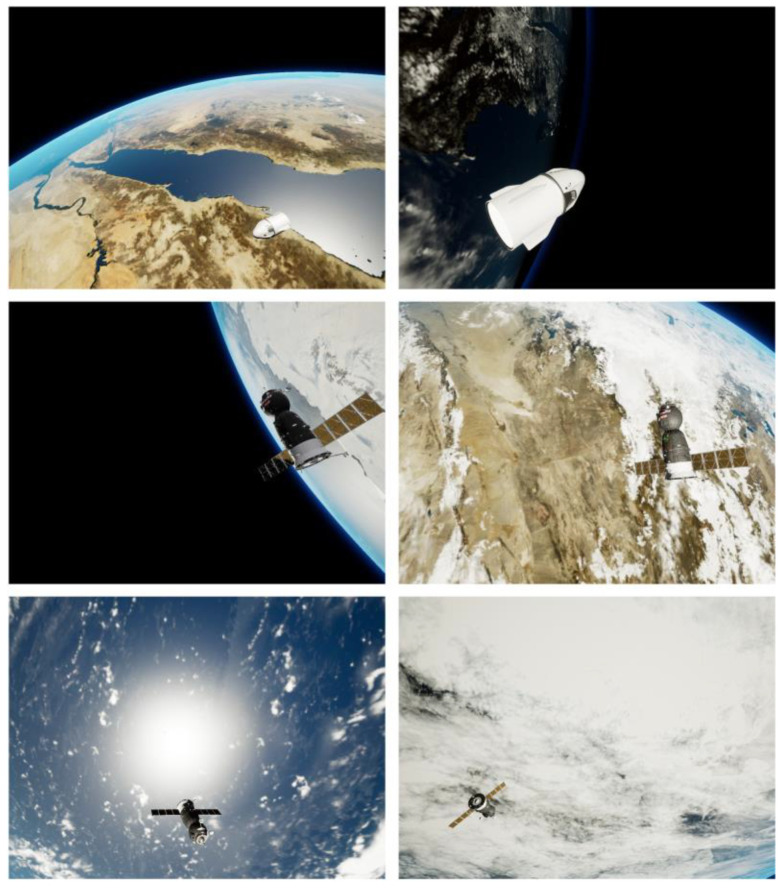
Sample figure of the URSO dataset [21].

Proenca et al. [21] conducted simulations involving two spacecraft, namely Soyuz and Dragon, on Unreal Engine 4. They proceeded to construct an URSO dataset, proposed a deep learning framework for orientation-based soft classification for bit pose estimation, and used ResNet as the backbone network to directly regress the output pose. This was achieved by extracting bottleneck features and minimizing the relative error, which served as the loss function. This paper conducts relevant research through the URSO dataset, which uses multiple satellite models, providing a robust foundation for assessing the model’s universality. In addition, this dataset uses Unreal Engine 4 to render rich space backgrounds and restore as many real-world space environments as possible, as shown in Figure 1. While these complex backgrounds simulate the real space environment, they can significantly impact the accuracy of satellite pose estimation.

Since the introduction of transformers [22] in the field of machine translation in 2017, these models have been widely used in various fields, including natural speech processing, speech recognition, and image processing [23,24]. At the heart of transformers lies the self-attention mechanism, which mimics the human eye’s ability to focus on essential information while suppressing irrelevant details. This mechanism possesses remarkable capabilities for both local feature extraction and long-range feature associations, amalgamating the strengths of convolutional neural networks and recurrent neural networks. Vision transformer (ViT) [25] was proposed by Dosovitskiy et al. in 2020, marking the first instance of incorporating transformer architecture into the field of computer vision. ViT has since gained considerable traction in downstream tasks [26] like image classification, object recognition, image segmentation, object tracking, and 3D reconstruction. However, the use of transformers as the core module in the context of 6-DoF is rare. Addressing the challenge of how to reasonably design the transformer model to accomplish the pose estimation task remains an urgent and unresolved issue.

To address the aforementioned challenges, we designed a new end-to-end satellite pose estimation network, which can reason out the satellite pose information in a single step, simplifying the inference process. The network mainly comprises a CNN backbone module, a translation regression network, and an orientation regression network. The CNN backbone module loads the EfficientNet pretraining model and learns the data’s feature information. The translation regression network and orientation regression network are mainly composed of filterformer and fully connected layers, in which filterformer integrates the transformer encoder and filter design ideas and generates adaptive weights by using the hypernetwork-like class [27]. This approach outperforms conventional convolutional neural networks and other network architectures in the satellite pose estimation task. The main contributions of this paper are as follows:

- A new end-to-end satellite pose estimation network is proposed, which incorporates an improved transformer model and employs a dual-channel network structure. This effectively decouples the translation information and orientation information of the satellite objects;
- A filterformer network structure is designed, which effectively integrates the advantages of the transformer encoder and the filter, enabling the network to adapt to complex background environments and effectively improving the satellite pose inference accuracy;
- The effectiveness of the method is verified in different computer vision downstream tasks.

## 2. Related Work

This section primarily analyzes and compares various methods of satellite pose estimation networks and describes them through different processing processes. Furthermore, the development of transformers in the field of computer vision is examined and its advantages and disadvantages and its application in pose estimation are briefly described.

### 2.1. Satellite Pose Estimation Network

Satellite pose estimation based on monocular vision is mainly divided into two methods: the hybrid modular method and the direct end-to-end method. Among them, the direct end-to-end method is based on the deep learning model, which inputs the satellite image to be detected and directly infers the pose information of the satellite object in the image through the model. The hybrid module method mainly consists of three components: (1) spacecraft localization; (2) key point detection; and (3) pose calculation. In the spacecraft localization stage, the deep learning object detection framework can be used to detect the satellite, and the pose of the satellite can be accurately identified by predicting the bounding box around the object satellite. These bounding boxes are then used to select regions of interest (RoI). Essentially, this step involves cropping out specific portions of the image that contain the spacecraft, extracting the RoI through processing and analyzing the shape, texture, and other visual features to infer key information about the spacecraft. Finally, the satellite’s pose information is determined by PnP and RANSAC.

#### 2.1.1. Direct End-to-End Approaches

In the end-to-end satellite pose estimation task, most of the current inference models are improved based on the CNN architecture, which has a powerful local feature extraction capability and can extract rich semantic information from images. Here are a few notable examples: Proença et al. [21] proposed URSONet, featuring a ResNet-based backbone architecture. This model is divided into two branches, each dedicated to reasoning about translation and orientation information, respectively. A categorical continuous orientation estimation method based on soft-assignment coding is also proposed, where each ground truth label is coded as a Gaussian random variable in the discrete output space of the orientation. The network is then trained to output a probability mass function that corresponds to the actual orientation. Albert et al. [28] proposed a CNN that directly regresses the pose without any a priori 3D information in a simple yet effective way of predicting the bounding box of the spacecraft in the image; they combined the predicted localization into a center detection network, performing ROI cropping and using it as an input for orientation estimation, thus reasoning about the pose information of the satellite using denser and richer features. Park et al. [29] proposed a spacecraft pose estimation method called SPNv2, which aims to bridge the domain gap between synthetic training images and spaceborne test images. The proposed method utilizes a multi-scale, multi-task CNN architecture and incorporates extensive data augmentation and domain randomization for robust training.

#### 2.1.2. Hybrid Modular Approaches

Chen et al. [30] proposed a method for estimating the 6-DOF pose of a satellite relative to the canonical pose from a single image. This method combines machine learning and geometric optimization by first localizing the satellite object through an object detection algorithm and then detecting the key points of the satellite object through a high-resolution network (HRNet); it then correlates the key points with the 3D points on an a priori reconstructed 3D model. Finally, the object’s pose is determined through a process of nonlinear optimization. Kiruki et al. [31] used YOLO for satellite object detection, provided the cropped RoI to the U-Net network for key point prediction, and used PnP and RANSAC for pose inference. The model was deployed into an FPGA with the CPU hardware, and the performance of the reasoning between the onboard computer and the ground computer was compared. Kecen et al. [32] proposed a learning-based pose estimation method for spacecraft using satellite images. The method utilizes a Keypoint Detection Network (KDN) to detect and localize keypoints on the spacecraft. The 3D coordinates of the keypoints are then estimated using a PnP algorithm. The method also incorporates an uncertainty prediction strategy to select more accurate keypoints for pose estimation. Wang et al. [33] proposed the use of transformer models in monocular satellite pose estimation. The proposed method utilizes transformer blocks to predict keypoints on the satellite. They address the limitations of convolutional neural networks in capturing long-range dependencies and generalizing to new datasets. The proposed approach achieves reliable pose estimation and better generalization performance compared to comparable methods.

### 2.2. Transformer

The transformer framework stands as a prominent focus of contemporary research, with the Vision Transformer (ViT) emerging as a significant breakthrough in recent years within the field of computer vision. ViT is trained by partitioning images and feeding them into the transformer module through a series of dimensional transformations. However, ViT exhibits suboptimal performance when applied to small datasets compared to traditional convolutional neural networks of the same size. However, ViT achieves remarkable results on large datasets. This result is somewhat predictable since the transformer lacks the inherent translation invariance of convolutional structures and the ability to learn local features, making it difficult to fit data efficiently with a small amount of data. In response to this challenge, researchers have introduced various techniques aimed at enhancing ViT’s performance. Among them, the Swin transformer has emerged as a frontrunner, surpassing traditional convolutional neural networks across a range of tasks and delivering exceptional results. It achieves this by reducing the number of parameters by segmenting the image into small windows, using the local self-attention mechanism, and realizing information exchange through window sliding.

A 6-DoF bit-pose estimation network based on a ViT module, 6D-ViT [34], has two ViT channels that extract RGB features and point-cloud features, respectively, to semantically enhance the quality of the instance representations by capturing the long-range contextual dependencies of the elements from the RGB image and the point cloud. Other studies have fused convolutional networks with transformers; for example, transformer pose (TFPose) uses a convolutional neural network as a backbone network to extract image features and then inputs them into the transformer module for key point prediction. Then, they use PnP and RANSAC for pose solving. In this paper, we draw inspiration from the concept of employing a dual-channel configuration and CNN as the foundational network for feature extraction. However, the distinction lies in how FilterformerPose utilizes a dual-channel filterformer to extract RGB image features and independently deduce the object’s translation and orientation information. On the other hand, the High-Resolution Transformer (HRFormer) is based on the HRNet backbone. While HRNet primarily conducts feature extraction through a series of CNNs, HRFormer takes a different approach by replacing the primary CNN component with a transformer. Simultaneously, inspired by the Swin transformer concept, we divide the feature map into windows for self-attention learning, effectively reducing the number of parameters. This innovative approach has yielded favorable results.

## 3. FilterformerPose

The proposed FilterformerPose mainly consists of the following components: a CNN backbone loaded with Efficientnet pre-trained weights, a filterformer-based translation regressor, and an orientation regressor. The model utilizes the CNN backbone based on the Efficientnet [35] model for satellite image feature learning, which learns image features at various scales through a multilayer CNN structure. Furthermore, the model decouples the satellite’s translation and orientation information through the use of a two-channel filterformer network.

**Figure 2:**
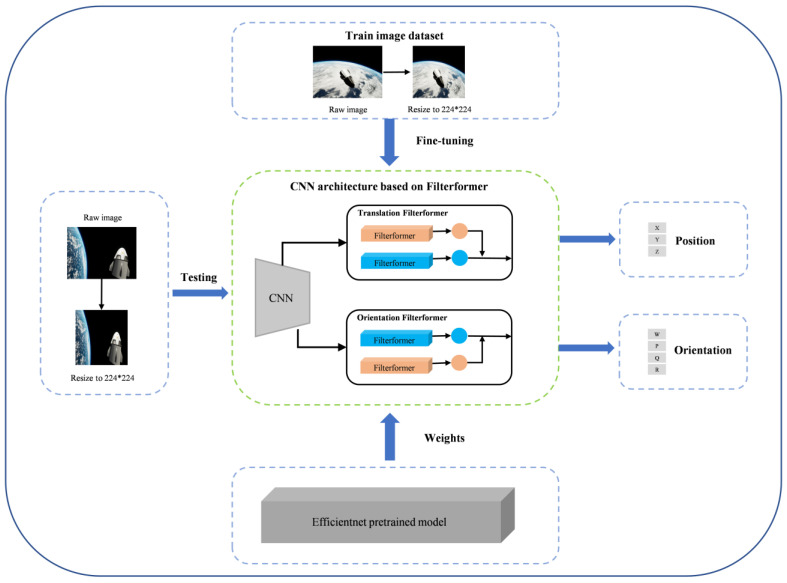
Flow chart of satellite pose estimation based on FilterformerPose network.

During the training process, the image was resized to 224 × 224. The network was loaded with Efficientnet pre-training weights, which were then fine-tuned with the training set. Following feature extraction of the data by the CNN backbone, two layers of features were randomly extracted and input to the translation filterformer and orientation filterformer. These models predicted inference results, calculated loss distance with the real label, and used gradient descent to complete iterative training until the optimal solution was reached. After obtaining the optimal model, the test set was processed according to the same data processing method as the training set, and the optimal model was loaded to perform satellite translation information reasoning to assess the model’s effectiveness. The flow is shown in Figure 2.

### 3.1. Network Architecture

**Figure 3:**
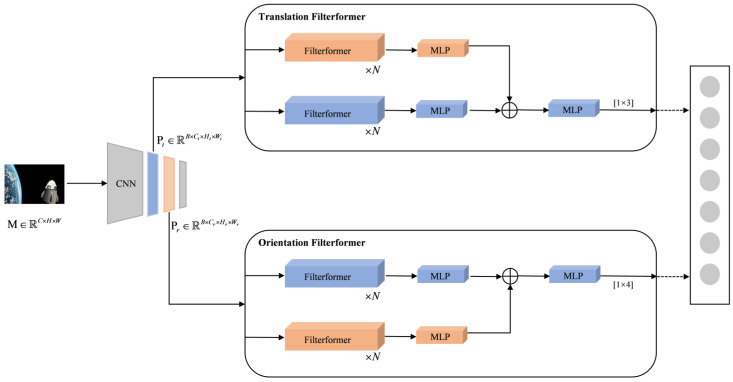
FilterformerPose satellite pose estimation network.

The two-channel FilterformerPose is shown in Figure 3; given a satellite image $P\in {\mathbb{R}}^{C\times H\times W}$, the input batch size is B and the EfficientNet pre-training model is loaded. After using the CNN Backbone feature extraction network, two different feature layers $P_{t}\in {\mathbb{R}}^{14\times 14\times 112}$ and $P_{r}\in {\mathbb{R}}^{28\times 28\times 40}$ were selected [36] and input to the translation filterformer and the orientation filterformer, comprising two regression sub-networks for decoupled learning. Each branch consists of filterformers and multi-layer perception (MLP). Finally, feature fusion is performed using the hypernetwork idea. To ensure compatibility with the inputs of the filterformer model, $P_{t}$ and $P_{r}$ need to be correlated using 1 × 1 convolution and flattening according to certain rules and transformed into $\overset{⌢}{P}\in {\mathbb{R}}^{B\times X\times Y}$. After the features enter the bit-pose regression sub-network, the process unfolds in two main steps. Firstly, feature learning is performed using the filterformer, followed by feature solving using MLP. In the bit-pose regression sub-network, a class hyper-network design is introduced where one of the branches generates hyper-parameters, and an optimization algorithm is used to iteratively search for the best combination of hyper-parameters in the hyper-parameter space to optimize the performance of the pose regressor. Following MLP feature fusion, the features are fed into the regression sub-network MLP inference header. The dimension of this MLP inference header is determined by the number of labels. Specifically, in the translation filterformer, the dimension number is 3, while in the orientation filterformer, the dimension number is 4.

**Figure 4:**
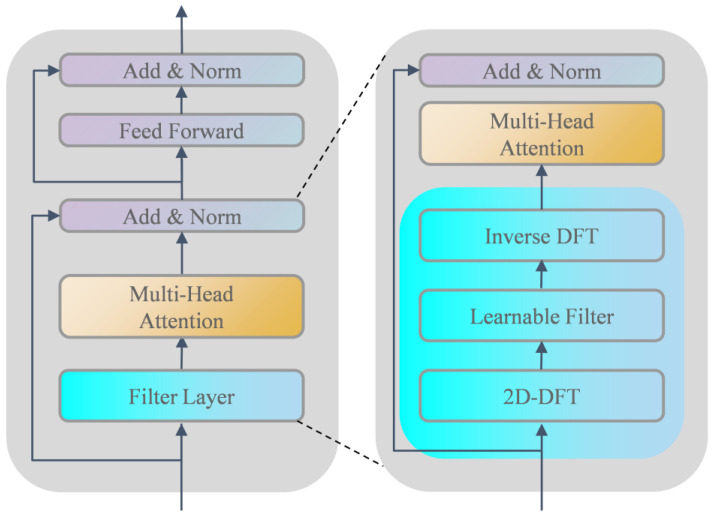
Schematic diagram of the filterformer structure.

Filterformer is primarily based on transformer encoder architecture to design a filterformer with a filtering function. Its structure is shown in Figure 4. The network consists of several blocks including a filter layer, self-attention mechanism (SA), Multi-Head Attention (MHA), residual connection and layer normalization (LN) block, and feed forward network (FFN). Within this framework, the self-attention mechanism serves as the fundamental building block of MHA.

#### 3.1.1. Filter Layer

Two-dimensional discrete Fourier transform (2D-DFT) is a digital transformation method whose main function is to transfer an image from the spatial domain to the frequency domain. First, the features are operated in 2D-DFT, and the features in each dimension are filtered in the frequency domain:

(1) $$Q^{l}=\Gamma (P^{l})\in {\mathbb{R}}^{B\times X\times Y}$$

Γ(⋅) represents 2D-DFT, and $Q^{l}$ represents the spectrogram of $P^{l}$. We can modulate the spectral characteristics by setting a learnable parameter, which operates as follows:

(2) $${\hat{Q}}^{l}=W⊙Q^{l}$$

⊙ is element-by-element multiplication and W is the learnable weight matrix. The introduction of W can dynamically learn the frequency feature and make this part differentiable, so as to realize its iterative training on the network.

Finally, the inverse two-dimensional discrete Fourier transform (Inverse 2D-DFT) is used to transform spectrum ${\hat{Q}}^{l}$ back into time domain ${\hat{P}}^{l}$ and update the sequence representation:

(3) $${\hat{P}}^{l}=\Gamma^{-1}({\hat{Q}}^{l})\in {\mathbb{R}}^{B\times X\times Y}$$

$\Gamma^{-1}(\cdot )$ represents the inverse 2D-DFT, which converts the spectrogram after passing through the learnable layer into a time domain map. Through the operation of 2D-DFT and Inverse 2D-DFT, the background noise in the image features can be effectively removed. This results in an improved quality of the features that are fed into the Multi-Head Attention (MHA) module.

#### 3.1.2. Self-Attention

**Figure 5:**
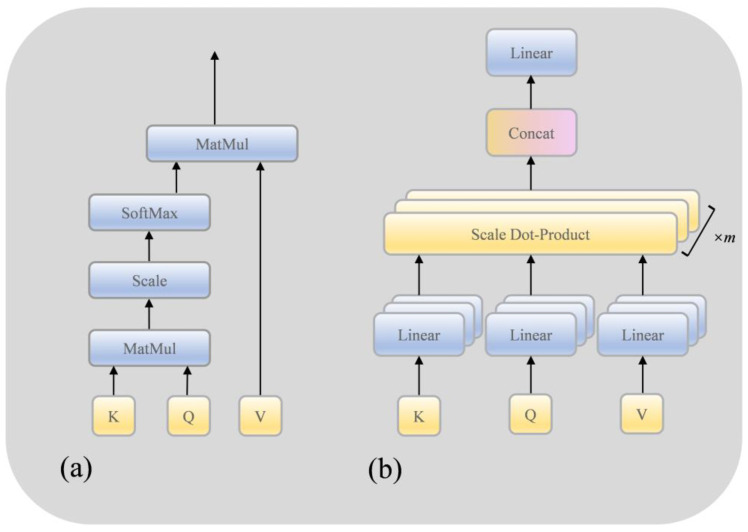
Structure diagram of SA (**a**) model and MHA (**b**) model.

The self-attention mechanism is a crucial technology applied in both natural language processing and computer vision. Its fundamental concept involves allowing each element within the input sequence to interact with all others, assigning weights to each element based on the results of these interactions. In this way, the self-attention mechanism is able to capture the relationships between different elements in the input sequence and generate a context-sensitive representation. By establishing these interaction connections among elements, the self-attention mechanism effectively captures long-range dependencies, offering the benefits of robust parallel computing capabilities and interpretability. As shown in Figure 5a, the self-attention mechanism is mainly implemented by scaling the dot product,

(4) $$A\mathrm{ttention}(Q,K,V)=soft\max(\frac{QK^{T}}{\sqrt{d_{k}}})V$$

where Q, K and V represent the query matrix, key matrix, and value matrix, which are obtained by multiplying the feature matrix and three random weight matrices, respectively, and $d_{k}$ is the dimension of the input feature.

#### 3.1.3. Multi-Head Attention

MHA is an extended form of the self-attention mechanism, as shown in Figure 5b. The expression ability and learning ability of the model are enhanced by introducing multiple attention heads. Each attention head can learn a different weight distribution to capture different levels and different types of attention patterns. Its expression is as follows:

(5) $$head_{i}=A\mathrm{ttention}(QW_{i}^{Q},KW_{i}^{K},VW_{i}^{V})$$

where $W_{i}^{Q},W_{i}^{K}\in {\mathbb{R}}^{d_{\mathrm{model}}\times d_{k}}$, $W_{i}^{V}\in {\mathbb{R}}^{d_{\mathrm{model}}\times d_{v}}$, $d_{\mathrm{model}}$ represent the length of the input features, where $d_{k}=d_{v}=d_{\mathrm{model}}/h$, h represents the number of heads. Splice the result of the projection calculation of multiple heads:

(6) $$MHA(Q,K,V)=Concat(head_{1},head_{2},\cdots ,head_{h})W^{o}$$

where $W^{O}\in {\mathbb{R}}^{hd_{v}\times d_{\mathrm{model}}}$.

Each filterformer layer contains a feed-forward neural network that performs a nonlinear transformation of the output of the self-attention mechanism. A feedforward neural network consists of two fully connected layers with nonlinear transformations through activation functions such as ReLU between them. The function of the feedforward neural network is to independently map and extract features from the representation of each location and enhance the expression ability of the features learned by the model in the sequence. To avoid issues like gradient disappearance or explosion, particularly when the model involves numerous layers, the transformer framework introduces residual connections between the inputs and outputs of each sublayer (including the Filter Layer, SA, and FFN). The residual join directly adds the representation of the input to the representation of the output of the sublayer, preserving the input information and allowing the gradient to flow better when backpropagated. Residual joins help improve the training effectiveness and optimization ability of the model.

### 3.2. Quaternion Activation Function

The neural network adds activation functions at neurons to enhance the model expression ability, and the features are nonlinearly processed to enhance the model expression richness. However, in the URSO dataset, the orientation of the satellite pose is represented by the quaternion $\left[q_{0},q_{1},q_{2},q_{3}\right]$, and there is an associated constraint on the quaternion; that is,

(7) $$q_{0}{}^{2}+q_{1}{}^{2}+q_{2}{}^{2}+q_{3}{}^{2}=1$$

The conventional activation function cannot meet the characteristics of this labeling information. In order to make the output information of the model predicted orientation satisfy Equation (7), normalization [37] is used with the expression:

(8) $$f(q)=\frac{q_{i}}{\sqrt{\sum_{i=0}^{n}q_{i}}}(n=3)$$

This activation function outputs four values, and the output is consistent with the satellite pose orientation information.

### 3.3. The Joint Loss Function of Pose

The loss value of the model is described by calculating the distance between the amount of translation and orientation of the true value $\langle t_{gt},q_{gt}\rangle$ and the predicted value $\langle t_{est},q_{est}\rangle$. The $t\in {\mathbb{R}}^{3}$ represents the spatial translation of the object relative to the camera, and $q\in {\mathbb{R}}^{4}$ represents the orientation information. A pose joint loss function [38] is used, which consists of a translation loss function and an orientation loss function; it then controls the balance of the two loss functions by the learning parameters, where the translation loss function is,

(9) $$L_{t}={\left\|t_{gt}-t_{est}\right\|}_{2}$$

the orientation loss function,

(10) $$L_{q}={\left\|q_{gt}-\frac{q_{est}}{\left\|q_{est}\right\|}\right\|}_{2}$$

the joint loss function of pose,

(11) $$L_{loss}=L_{t}\exp(-s_{t})+s_{t}+L_{q}\exp(-s_{q})+s_{q}$$

where $s_{t}$ and $s_{q}$ are learning parameters used to adjust the translation and orientation loss function weighting coefficients.

## 4. Experimental Results

### 4.1. URSO Analysis

URSO is a simulator built based on Unreal Engine 4, which is used to render realistic images of spacecraft within Earth’s orbit. By harnessing the robust graphics rendering capabilities and physics simulation functionalities inherent in Unreal Engine 4, URSO excels at producing realistic lighting, shadows, materials, and textures. Consequently, the spacecraft rendered through URSO exhibits a high level of visual authenticity. By using URSO for simulation and evaluation, we can avoid the high costs, risks, and restrictions associated with conducting experiments directly in the actual space environment. The URSO dataset uses two kinds of satellites, namely, Soyuz and Dragon, and is divided into three sub-datasets according to the distance between the object and the observation point, namely, soyuz_easy, soyuz_hard, and dragon _hard.

URSO provides labeling information, which mainly contains the translation and orientation of the object satellite in the format $\left[q_{0},q_{1},q_{2},q_{3},x,y,z\right]$. The quaternion $\left[q_{0},q_{1},q_{2},q_{3}\right]$ represents the amount of orientation and [x,y,z] represents the amount of translation. Quaternions offer an elegant and stable solution for representing orientation, effectively avoiding the gimbal lock problem in certain scenarios. However, when a human-readable description is needed, Euler angles may be a more suitable choice for visualizing orientation. Equation (12) is used to convert quaternions into Euler angles, and the resulting yaw, pitch, and roll represent the yaw, pitch, and roll angles of the object satellite, respectively.

(12) $$\left[\begin{array}{c} R_{roll}\\ R_{pith}\\ R_{yaw} \end{array}\right]=\left[\begin{array}{c} \phi \\ \theta \\ \psi \end{array}\right]=\left[\begin{array}{c} \arctan\frac{2(q_{0}q_{1}+q_{2}q_{3})}{1-2(q_{1}{}^{2}+q_{2}{}^{2})}\\ \arcsin(2(q_{0}q_{2}+q_{1}q_{3}))\\ \arctan\frac{2(q_{0}q_{3}+q_{1}q_{2})}{1-2(q_{2}{}^{2}+q_{3}{}^{2})} \end{array}\right]$$

**Figure 6:**
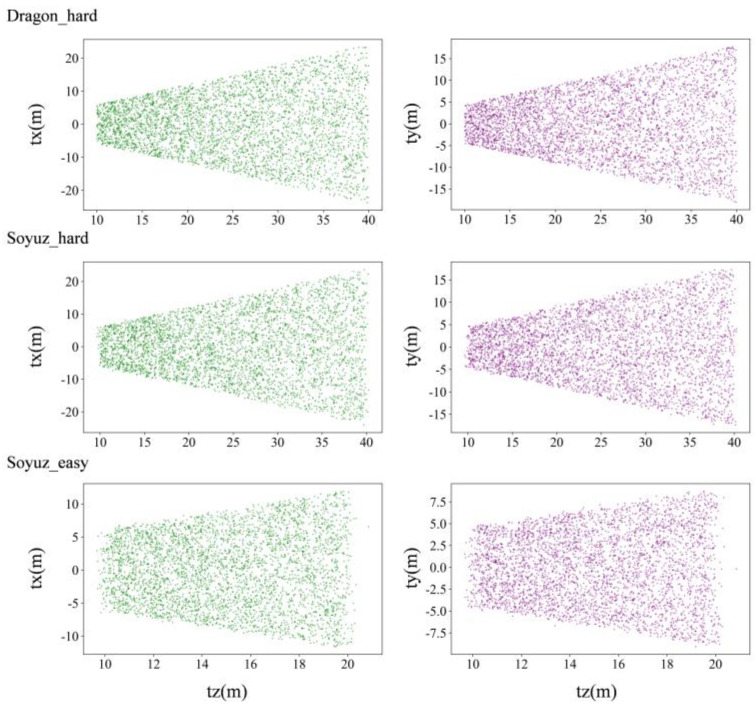
Distribution of translation of URSO dataset.

**Figure 7:**
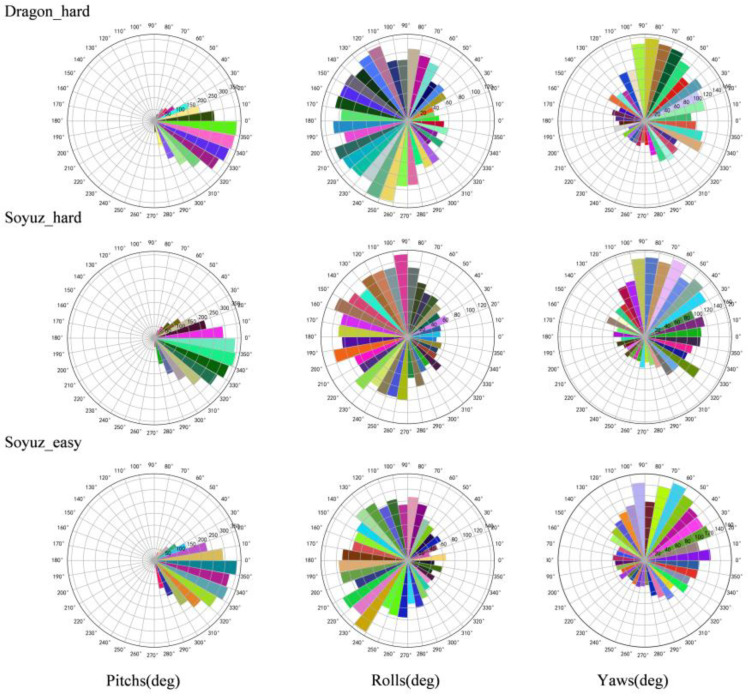
Distribution of orientation of URSO dataset.

A visualization method is used to better clarify the distribution of satellite pose in the labels, whose translation and orientation information are shown in Figure 6 and Figure 7, respectively.

As can be seen from Figure 6 and Figure 7, soyuz_easy is mainly distributed in the range of 8~22 m from the observation point, 0~12 m up and down; soyuz_hard is mainly distributed in the range of 8~42 m from the observation point, 0~22 m up and down; and dragon_hard is mainly distributed in the range of 8~42 m from the observation point, 0~22 m up and down. In the orientation angle, the distribution across all three datasets is relatively consistent, the pitch angle is in the range of −60°~60°, and the yaw and roll angles cover all angles. Through an analysis of the pose distribution within the URSO dataset and its alignment with tasks related to satellite pose estimation, it becomes evident that the dataset’s distribution aligns with the requirements of these tasks. It boasts a wide-ranging data distribution, which serves as strong support for real-world applications within this scenario.

### 4.2. Evaluation Metrics

In this experiment, in addition to satellite pose experiments, camera pose localization experiments will also be performed using the Cambridge Landmarks dataset [39]. While pose estimation and camera pose localization are distinct downstream tasks, they share common physical information and evaluation metrics. Both tasks utilize Absolute Pose Regression as an evaluation criterion.

Taking the Euclidean distance error $E_{t}$ between the actual translation $t_{gt}$ and the predicted translation $t_{est}$ as an evaluation measure, the accuracy of relative translation regression is analyzed, and the calculation formula is as follows:

(13) $$E_{t}={\left\|t_{gt}-t_{est}\right\|}_{2}$$

Taking the axis angle error $E_{q}$ between the actual pose $q_{gt}$ and the predicted pose $t_{est}$ as the evaluation index, the accuracy of the relative pose prediction is analyzed, and the calculation formula is as follows:

(14) $$E_{q}=2\cos^{-1}\left|\langle q_{gt},q_{est}\rangle \right|$$

### 4.3. Training Details

The deep learning framework of PyTorch and Pycharm (Python 3.8) with Anaconda 4.12.0 was used as the simulation platform. The hardware configuration was i9-11900 K 3.50 GHz with 64 G RAM for CPU and NVIDIA RTX3090 with 24 G of memory for GPU. Our model was trained in three stages. First, the entire network was trained iteratively for 500 epochs. Then, the orientation filterformer was frozen, fine-tuned using the translation filterformer, and trained iteratively for 150 epochs. Finally, the translation filterformer is frozen, and fine-tuned using the orientation filterformer for fine-tuning and iterative training for 150 epochs. For the network hyper-parameter settings, the learning rate was 10^−4^, and the learning parameters in the loss function were $s_{t}=-3$ and $s_{q}=-6.5$. The data augmentation methods used during the training process of the network include resizing images, random cropping, and adjustments to brightness, contrast, and saturation. During the testing process, the images are processed by scaling and center cropping, without any other augmentation operations. During training and testing, the images were scaled and adjusted to 224 × 224 in order to facilitate the network input. 

### 4.4. Ablation Study

To assess the performance of the selected structures, we conducted an ablation study, focusing on the Soyuz_hard scene within the URSO dataset. The object detection task in the Soyuz_hard scene was identical to that in the Soyuz_easy scene. In addition, the detection distance and orientation of the Soyuz_hard scenario were identical to that of the Dragon_hard scenario, which is representative of this design. For each ablation, only one single parameter was changed. In our experiments, we first performed the filterformer component number of 5 and different MLP dimension number experiments. Once the optimal number of MLP dimensions was determined, we then identified the optimal number of filterformer components by experimenting with different numbers of them. Lastly, we conducted experiments to assess the impact of the filter layer on the network across all three datasets.

#### 4.4.1. MLP Dimensions

In this experiment, the number of filterformer components was set to N=5. The number of dimensions of the MLPs of the two regression sub-networks, translation filterformer, and orientation filterformer were varied separately when performing the ablation experiments. We conducted experiments with dimension numbers of 128/128, 128/256, and 256/256. For example, 128/256 means that the number of MLP dimensions in the translation filterformer is 128, and the number of MLP dimensions in the orientation filterformer is 256. The results of the experiments are shown in Table 1.

Upon analyzing and comparing the experimental results, it is observed that the best orientation accuracy is achieved when the number of dimensions is set to 128/256. Conversely, the best translation accuracy is obtained when the number of dimensions is 128/256 as well. To strike a balance between model size and inference accuracy, we have opted for 128/256 as the number of dimensions for the MLP.

#### 4.4.2. The Number of Filterformer Components

The number of filterformer components in FilterformerPose can be adjusted based on task requirements. Using different numbers of filterformer components can impact the model’s inference accuracy. Through experimental analysis and comparison, the best filterformer numbers were selected. At the same time, we count the inference time of a single image with different numbers of filterformer components.

Ablation experiments were carried out using 3–9 filterformers, and all experimental conditions remained the same except for the different numbers of filterformers. The results are presented in Table 2. It can be observed that as the number of filterformers increases, when N∈(3,6), its translation and orientation errors decrease. When N∈(7,9), its translation errors and orientation errors tend to level off. In order to balance the model size and reasoning accuracy, N=7 was chosen as the optimal number of filterformers.

#### 4.4.3. Filter Layer

The ablation experiment was added to verify the beneficial effect of the filter on the network model. Comparison experiments were conducted using a network without the filter layer, which was named TransformerPose. The results of the experiments are shown in Table 3. On the Soyuz_hard and Dragon_hard datasets, TransformerPose exhibits slightly better translation accuracy compared to FilterformerPose. However, TransformerPose lags behind FilterformerPose in other metrics, particularly in orientation accuracy, where it falls significantly short of FilterformerPose.

### 4.5. Results

**Figure 8:**
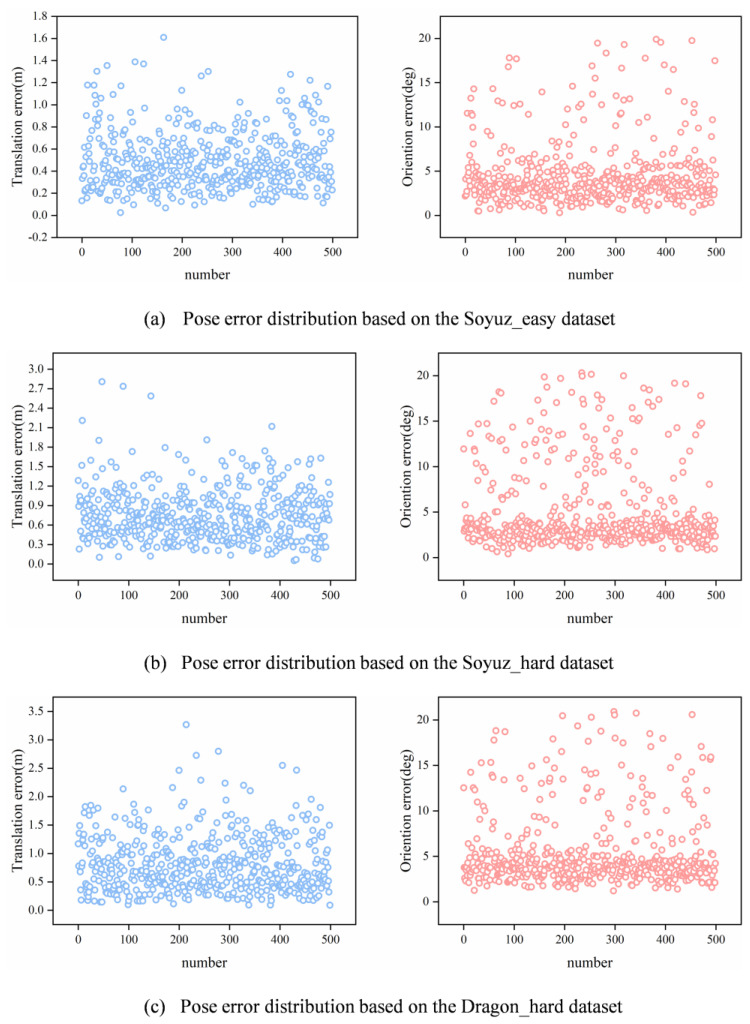
Pose error distribution based on the URSO dataset.

For the experiments, the optimal number of filterformers was selected as N=7 and MLP with dimensions 128/256. As shown in Figure 8a, the orientation error of FilterformerPose in the Soyuz_easy dataset is within 20°, which is mainly concentrated within 5°. The translation errors are all within 1.6 m, and most of them are within 0.6 m. As shown in Figure 8b, the orientation error of FilterformerPose in the Soyuz_hard dataset is within 20°, of which it is mainly concentrated within 5°; the translation error is within 2.8 m, most of which are within 0.9 m. As shown in Figure 8c, the orientation error of FilterformerPose in the Dragon_hard dataset is within 21°, of which it is mainly concentrated within 5°; the translation error is within 3.3 m, most of which is within 0.9 m.

Comparing the pose estimation accuracy of the proposed method with that of UrsoNet based on the URSO dataset, as shown in Table 4, it is evident that the pose estimation accuracy of the proposed method achieves better results on each dataset. Among them, the translation accuracy improved compared to the UrsoNet method, and orientation errors reduced from 13.9480 to 5.5256 when reasoning about the more complex dataset (Dragon_hard), which is more significant. Through the above analysis, the model’s accuracy for orientation saw a significant improvement on the URSO dataset, and its effect is even more prominent on complex object models and long-range objects.

### 4.6. Evaluation of Cambridge Landmarks

Finally, to demonstrate that our approach can be adapted to different downstream tasks, we conducted relevant experiments on the Cambridge Landmarks dataset, a publicly available dataset in the field of camera pose localization, which plays a pivotal role in computer vision and augmented reality by furnishing essential information about the camera’s translation and orientation in 3D space. Such information serves as the fundamental support for many applications. We trained separate models for each outdoor scene within the Cambridge Landmark dataset.

Compared with other methods, FilterformerPose achieved remarkable results, particularly in translation accuracy, and was second only to the MS-Trans [36] method in orientation accuracy, as shown in Table 5. These results demonstrate that our method can be used in the downstream task of camera pose localization with accuracy, comparable to current advanced methods.

## 5. Conclusions

A novel satellite pose estimation model based on filterformer was proposed to address the problem of estimating the pose of non-cooperative objects in space. Compared to the existing direct end-to-end methods, FilterformerPose offers distinct advantages. It excels at capturing long-distance dependencies, enabling comprehensive understanding of spatial relationships. Additionally, it effectively extracts local features by selectively attending to relevant contextual information. The fusion of these abilities significantly contributes to the improved performance of FilterformerPose. The model uses a two-channel network design to decouple the translation information and orientation information of the object. By designing a filter-based transformer encoder model and constructing a filter self-attention mechanism-based hypernetwork-like design, it was possible to achieve effective noise reduction and generate adaptive weight information. To evaluate the performance of our approach, we conducted experiments on the URSO satellite pose estimation dataset. The results of these experiments demonstrate that the inclusion of the filter layer in our network significantly enhances pose estimation accuracy. Meanwhile, FilterformerPose has a better effect on orientation accuracy enhancement, especially on the Dragon_hard dataset with complex object models and long-distance objects. FilterformerPose reduced the orientation error from 13.9480 to 5.5256. Related experiments have demonstrated the potential applications of FilterformerPose in satellite pose estimation tasks.

FilterformerPose exhibits adaptability to diverse computer vision tasks and has demonstrated remarkable performance in camera pose localization datasets. This capability holds significant implications for space missions, including relative navigation, virtual reality space capsule control, and space target tracking, where accurate camera pose localization is pivotal.

## Figures and Tables

**Table 1:** Comparison of pose accuracy for different MLP dimensions.

MLP Dimensions (Translation/Orientation)	Translation (Meters)	Orientation (Degrees)
128/128	0.8956	6.3023
128/256	0.8735	5.8025
256/256	0.8546	6.1258

**Table 2:** Comparison table of different filterformer magnitude pose accuracy.

Filterformer Numbers	Translation (Meters)	Orientation (Degrees)	Inference Time (ms)
N = 3	1.0125	6.5437	61.61
N = 4	0.9233	5.9961	64.89
N = 5	0.8735	5.8025	68.11
N = 6	0.8155	5.3566	71.46
N = 7	0.7541	5.2023	74.73
N = 8	0.7623	5.3562	78.01
N = 9	0.7496	5.3534	81.49

**Table 3:** Comparison table of TransformerPose and FilterformerPose pose accuracy.

Dataset	TransformerPose (Translation/Orientation)	FilterformerPose (Translation/Orientation)
Soyuz_easy	0.5103 m, 5.1259°	0.4918 m, 4.6122°
Soyuz_hard	0.7283 m, 6.3654°	0.7541 m, 5.2023°
Dragon_hard	0.8076 m, 7.9168°	0.8160 m, 5.5256°

**Table 4:** Comparison table of TransformerPose and UrsoNet pose accuracy.

Dataset	Method	Translation (Meters)	Orientation (Degrees)
Soyuz_easy	UrsoNet	0.5288	5.3342
Soyuz_easy	FilterformerPose (ours)	0.4918	4.6122
Soyuz_hard	UrsoNet	0.8133	7.7207
Soyuz_hard	FilterformerPose (ours)	0.7541	5.2023
Dragon_hard	UrsoNet	0.8909	13.9480
Dragon_hard	FilterformerPose (ours)	0.8160	5.5256

**Table 5:** Comparison to state-of-the-art methods: Cambridge Landmarks.

Method	K.College	Old Hospital	Shop Facade	St.Mary	Avg.	Ranks
PoseNet [39]	1.92 m, 5.40°	2.31 m, 5.38°	1.46 m, 8.08°	2.56 m, 8.48°	2.08 m, 6.83°	9/10
LSTM-PN [40]	0.99 m, 3.65°	1.51 m, 4.29°	1.18 m, 7.44°	1.52 m, 6.68°	1.30 m, 5.57°	3/8
MapNet [41]	1.07 m, 1.89°	1.94 m, 3.91°	1.49 m, 4.22°	2.00 m, 4.53°	1.62 m, 3.64°	6/5
BayesianPN [42]	1.74 m, 4.06°	2.57 m, 5.14°	1.25 m, 7.54°	2.11 m, 8.38°	1.91 m, 6.28°	7/9
SVS-Pose [43]	1.06 m, 2.81°	1.50 m, 4.03°	0.63 m, 5.73°	2.11 m, 8.11°	1.32 m, 5.17°	4/7
GPoseNet [44]	1.61 m, 2.29°	2.62 m, 3.89°	1.14 m, 5.73°	2.93 m, 6.46°	2.07 m, 4.59°	8/6
IRPNet [45]	1.18 m, 2.19°	1.87 m, 3.38°	0.72 m, 3.47°	1.87 m, 4.94°	1.41 m, 3.50°	5/4
CGA-PoseNet [46]	1.36 m, 1.85°	2.52 m, 2.90°	0.74 m, 5.84°	2.12 m, 2.97°	2.84 m, 3.39°	10/3
MS-Trans [36]	0.83 m, 1.47°	1.81 m, 2.39°	0.86 m, 3.07°	1.62 m, 3.99°	1.14 m, 2.73°	2/1
FilterformerPose (Ours)	0.67 m, 2.57°	1.88 m, 3.38°	0.73 m, 3.59°	1.08 m, 3.94°	1.09 m, 3.37°	1/2

## Data Availability

Not applicable.

## Abbreviations

CNNs	Convolutional neural networks
URSO	Unreal rendered spacecraft on-orbit
SFF	Spacecraft formation flying
OOM	On-orbit maintenance
ADR	Active debris removal
6-DoF	Six-degrees of freedom
PnP	Perspective-n-Points
RANSAC	Random sample consensus
SPN	Spacecraft pose network
RoI	Regions of interest
ViT	Vision transformer
TFPose	Transformer pose
HRFormer	High-resolution transformer
HRNet	High-resolution network
KDN	Keypoint Detection Network
MLP	Multi-layer perception
SA	Self-attention mechanism
MHA	Multi-head attention
LN	Residual connection and layer normalization
FFN	Feed forward network
2D-DFT	Two-dimensional discrete Fourier transform
Inverse 2D-DFT	Inverse Two-dimensional discrete Fourier transform
